# Mechanistic evidence that benzo[a]pyrene promotes an inflammatory microenvironment that drives the metastatic potential of human mammary cells

**DOI:** 10.1007/s00204-018-2291-z

**Published:** 2018-08-24

**Authors:** Durr-e-shahwar Malik, Rhiannon M. David, Nigel J. Gooderham

**Affiliations:** 1 Computational and Systems Medicine, Imperial College London, London, SW7 2AZ UK; 2 Present Address: Genetic Toxicology, Drug Safety and Metabolism, MSAS Unit, AstraZeneca, Cambridge, UK

**Keywords:** Benzo[a]pyrene, IL-6, TNF-α, Inflammation, MiRNA, Human mammary cells

## Abstract

Benzo[a]pyrene (B(a)P) is a major cancer-causing contaminant present in food such as cooked meats and cereals, and is ubiquitous in the environment in smoke derived from the combustion of organic material. Exposure to B(a)P is epidemiologically linked with the incidence of breast cancer. Although B(a)P is recognized as a complete genotoxic carcinogen, thought to act primarily via CYP-mediated metabolic activation to DNA-damaging species, there is also evidence that B(a)P exposure elicits other biological responses that promote development of the cancer phenotype. Here in mechanistic studies using human mammary cells MCF-7 and MDA-MB-231, we have explored mechanisms whereby B(a)P (10^− 8^ to 10^− 5^M) promotes inflammation pathways via TNF-α and NFκB leading to IL-6 upregulation, microRNA (Let7a, miR21 and miR29b) dysregulation and activation of VEGF. The miRNA dysregulation is associated with altered expression of inflammation mediators and increased migration and invasive potential of human mammary cancer cells. Our data suggest that mammary cell exposure to B(a)P results in perturbation of inflammation mediators and dysregulation of tumorigenic miRNAs, leading to an inflammation microenvironment that facilitates migration and invasion of mammary epithelial cells. These properties of B(a)P, together with its well-established metabolic activation to DNA-damaging species, offer mechanistic insights into its carcinogenic mode of action.

## Introduction

Cigarette smoke, of which benzo[a]pyrene (B(a)P) is a constituent, has been shown to stimulate the production of inflammation cytokines particularly in lungs (Lee et al. 2012; Lin et al. 2010; van der Vaart et al. 2004) and smoking is linked with increased risk of breast cancer (Cui et al. 2006). Moreover, intake of processed meat, known to be contaminated with B(a)P, increases circulating inflammation biomarkers in women (Ley et al. 2014) and consumption of a pro-inflammation diet can elevate the risk of breast cancer (Shivappa et al. 2015). This epidemiological and clinical evidence strongly implicate cigarette smoke and consumption of processed meat being linked with the increased incidence of breast cancer. Furthermore, extensive mechanistic and animal studies on the carcinogenic potential of B(a)P have led to IARC reporting that B(a)P, cigarette smoking and processed meat are all Class 1 category carcinogens (carcinogenic in humans) (http://monographs.iarc.fr/ENG/Monographs/vol100F/index.php).

Exposure to DNA-damaging environmental chemicals such as polycyclic aromatic hydrocarbons and food-derived carcinogens such as heterocyclic amines is strongly linked with the incidence of breast cancer (Armstrong and Doll 1975; McPherson et al. 2000), yet the mechanisms involved are only partially understood. B(a)P is a well-studied polycyclic aromatic hydrocarbon carcinogen that is a ubiquitous environmental contaminant with a well-understood ability to damage DNA (Courter et al. 2007). However, reports on the role of B(a)P in later stages of cancer, especially metastasis, are limited (Guo et al. 2015). Metastasis and invasion are the life-threatening oncogenic events in terminal cancer (Stracke and Liotta 1992). Metastasis is a multistage process with primary cancer cells invading the surrounding tissues, entering the blood circulation and establishing metastatic disease at distal sites (Stracke and Liotta 1992).

The role of inflammation in the incidence of cancer has long been known. Virchow hypothesized that cancer originates at the site of chronic inflammation that triggers cancer by increasing proliferation (Balkwill and Mantovani 2001; Coussens and Werb 2002). Tumor cells are dependent on the microenvironment for nourishment and growth, and the surrounding stroma comprises non-cancer cells (fibroblast, endothelial cells and various immune cells such as macrophages, neutrophils, mast cells and lymphocytes) and extracellular matrix (Li et al. 2007; Mbeunkui and Johann 2009). The secretion of various signaling molecules (cytokines, growth factors, and chemokines) by non-cancer cells surrounding the tumor promotes the survival of cancer cells by increasing angiogenesis. Cyclooxygenase-2 (COX-2) (Miller et al. 2005; Ristimaki et al. 2002; Singh et al. 2007), tumor necrosis factor-alpha (TNF-α) (Danforth and Sgagias 1996), vascular endothelial growth factor (VEGF) (Byrne et al. 2007) and IL-6 (Lin et al. 2015) are all inflammation mediators that play critical roles in metastasis and progression of breast cancer. Whether exposure to B(a)P has a role in the regulation of these inflammation mediators and contributes to these non-genotoxic events that can aggravate the metastatic potential of breast cancer cells is not clear.

Detection of DNA mutations in response to chemicals has facilitated risk assessment and improvement of environmental health (Pufulete et al. 2004); additionally, recent studies have shown that environmental chemicals can mediate epigenetic changes that can influence the status of various diseases (Baccarelli and Bollati 2009). It has also been shown that environmental agents such as pollution, cigarette smoke and food-derived carcinogens can influence the expression of miRNAs (Sonkoly and Pivarcsi 2011) associated with phenotype dysregulation and pathology. The use of miRNA profiles as an indicator of response to carcinogenic toxicants has been investigated (Gooderham and Koufaris 2014; Koufaris et al. 2012; Papaioannou et al. 2014).

In the present mechanistic study, we have examined the potential of B(a)P to regulate inflammation pathways and cancer-associated miRNA expression in mammary cells to understand the mechanisms behind BaP-mediated phenotype dysregulation and in particular the development of an inflammation environment.

We report that mammary cell exposure to B(a)P leads to altered expression of COX-2, TNF-α, VEGF, IL-6, and oncogenic miRNAs and collectively these interrelated events are consistent with B(a)P promoting an inflammation environment. Since inflammation is known to play critical roles in metastasis and progression of breast cancer, we suggest that this complement of B(a)P-mediated phenotypic responses promote and enhance the established genotoxic effects of B(a)P to thereby contribute to the potent carcinogenic potential of the compound.

## Methods

### Cell culture

The human breast adenocarcinoma MCF-7 (estrogen receptor α positive, ERα+) and MDA-MB-231 (estrogen receptor α negative, ERα−) cell lines were purchased from ATCC (LGC Prochem, Middlesex,UK) and were grown in minimum essential medium (MEM) (GIBO, Life technologies, Paisley, UK) supplemented with 10% fetal bovine serum (FBS), 100 units/ml of penicillin and streptomycin 100 µg and 2 mM l-glutamine. Cells were cultured routinely in 75-cm^2^ flasks in a humidified incubator at 37 °C, 5% CO_2_.

### Treatment

Prior to treatment, cells (MCF-7 and MDA-MB-231) plated at a density of 25,000 cells/well in 24-well plates were supplemented with 5% dextran-coated charcoal-stripped FBS (stripped media) for 72 h. Cells were treated with various concentrations of B(a)P (10^− 9^–10^− 6^ M) (Sigma-Aldrich) dissolved in dimethyl sulphoxide (DMSO) to give a concentration of 0.1% DMSO in the final incubate. In some experiments, cells were treated with NFkappaB (NFκB) inhibitor (25 µM, sc-3060) (Santa Cruz Biotechnology Inc) dissolved in RNAase/DNAase-free water. Treatment-induced cytotoxicity was determined by counting cells in a haemocytometer and assessing cell viability by TrypanBlue exclusion (GIBCO, Life technologies).

### Reverse transcription-quantitative-polymerase chain reaction (RT-qPCR)

Following treatment, cells were lysed using TRIzol reagent, chloroform (0.2 ml) added and samples centrifuged 12,000x*g* (10 min, 2–8 °C). The upper aqueous phase was transferred to a fresh tube and 5 µg of RNase-free glycogen (as carrier to aqueous phase) and 0.5 ml isopropyl alcohol was added and incubated (37 °C, 10 min) to precipitate RNA. Following incubation, cells were centrifuged at 12,000x*g* (10 min 2–8 °C). The gel-like pellet was washed with ethanol and re-dissolved in RNase-free water with heating (55–60 °C). Extracted RNA was quantified by UV spectroscopy (UV–Vis Nano-spectrophotometer, Implen, Essex, UK) and purity was assessed from 260/280 nm and 260/230 nm ratios. Reverse transcription (RT) of the extracted RNA (100–500 ng) was according to manufacturer protocol (Invitrogen). QPCR was performed using predesigned Taqman gene expression assays and FAST PCR master mix (Taqman, Applied Biosystems, Life technologies) using a StepOnePlus fast real-time PCR system (Applied Biosystems, Life technologies) according to the manufacturer’s protocol. Target gene expression was normalized to GAPDH expression and quantified using the delta-Ct method.

### Transfection with miRNA mimic

Transfection of cells with miRNA mimics was as previously described (Patel and Gooderham 2015). Briefly, cells (1 × 10^5^ cells/well) were seeded in 24-well plates and allowed to settle overnight in 10% FBS MEM medium (no penicillin/streptomycin). After overnight incubation, medium was replaced with 400 µl/well opti-MEM media (Gibco, Life Technologies), followed by the addition of 150 µl/well of Opti-MEM containing 2.5 µl of Lipofectamine 2000 reagent and 2.5 µl of 20 µM stock of miRNA mimic or miRNA negative control (Thermo Fisher Scientific, Cramlington, UK). Transfected MCF-7 and MDA-MB-231 cells were incubated at 37 °C, 5% CO_2_ for 24 h and 48 h, respectively, before harvesting RNA with TRIzol reagent (Invitrogen). Transfection efficiency was determined by co-transfection with FAM-labelled oligonucleotide and fluorimetric assessment of cellular internalization. Successful transfection of intact miRNA species was confirmed by qPCR after RNA isolation. The transfection procedure was optimized for MCF-7 and MDA-MB-231 as 24 and 48 h, respectively.

### Cell proliferation assay

Quantification of the viable cells was assessed with AlamarBlue (Invitrogen, Life technologies) according to the manufacturer’s protocol. Cells (5 × 10^4^ cells/well) were seeded in a 24-well plate. Healthy viable cells maintain a reducing potential within their cytosol. This cellular enzymic reducing activity converts AlamarBlue reagent into a detectable fluorescent product “resorufin”, which can be measured in a spectrofluorimeter. The fluorescence intensity using the AlamarBlue reagent was directly proportional to cell number. Briefly, Alamar Blue reagent (10% of final volume) was added to cells and incubated for 1 h at 37 °C, then fluorescence (excitation 560 nm/emission 590 nm) was read in a Fluostar plate reader (BMG Labtech). Results are expressed as fluorescence intensity of the test sample compared to the vehicle control.

### Wound-healing assay

MCF-7 and MDA-MB-231 cells were plated in 24-well plates (10^5^ cells/well) and were grown in 1 ml of 10% FBS media until confluent (72 h). Confluent cell sheets were then wounded in the shape of cross using a sterile tip, washed three times with PBS, and 1 ml of culture medium supplemented with 5% dextran-coated charcoal-stripped FBS was added to each well. Cells were then treated with B(a)P and digital pictures of the cell sheets were taken at 0, 24 and 72 h (10× magnification), a minimum of three pictures per wound channel. Wound width (channel) measurements were assessed in Image J program. The percentage migration was calculated as follows:

$$\% \,{\text{migration}}\,=\,{\text{Average}}\,{\text{of}}\,{\text{wound}}\,{\text{width}}\,{\text{at}}\,0\,{\text{h}}\,-\,{\text{average}}\,{\text{of}}\,{\text{wound}}\,{\text{width}}\,{\text{at}}\,t\;{\text{h}}/{\text{average}}\,{\text{of}}\,{\text{wound}}\,{\text{width}}\,{\text{at}}\,0\,{\text{h}}\, \times \,{\text{1}}00.$$

Results are presented as fold change compared to the vehicle control.

### Transwell migration and invasion assays

30,000 cells contained in 100 µl of culture media supplemented with 1% dextran-coated charcoal-stripped FBS media were added to the upper chamber of a 96-transwell insert system with 8-µm pores (BD Falcon, Oxford, UK). In the lower chamber, 100 µl of culture media supplemented with 10% FBS was added as chemoattractant. Cells were treated in the upper chamber and cultured for up to 72 h to encourage migration. Following culture, cells that had migrated to the lower chamber were isolated and viable cell number estimated by the Alamar Blue assay (see above) using addition of 10% AlamarBlue (Invitrogen, Life technologies) for 2 h at 37 °C. Results are expressed as fold change compared to the vehicle control. For the invasion assay, the upper chamber insert was coated with 20 µl of matrigel and allowed to settle at room temperature prior to addition of the cells. In this assay, migration to the lower chamber requires cells to digest their way through the matrigel layer. Migration of invasive viable cells was estimated using Alamar Blue assay as described above.

### Statistical analysis

To assess the statistical significance between different treatments, one-way analysis of variance (ANOVA) with Dunnet post-test was performed. Data were obtained from measurements made in at least three independent cultures and presented as a mean ± standard error (SEM) (GraphPad Prism 5, GraphPad Software Inc., La Jolla, CA, USA).

## Results

### Can B(a)P regulate the expression of inflammation mediators?

**Fig. 1:**
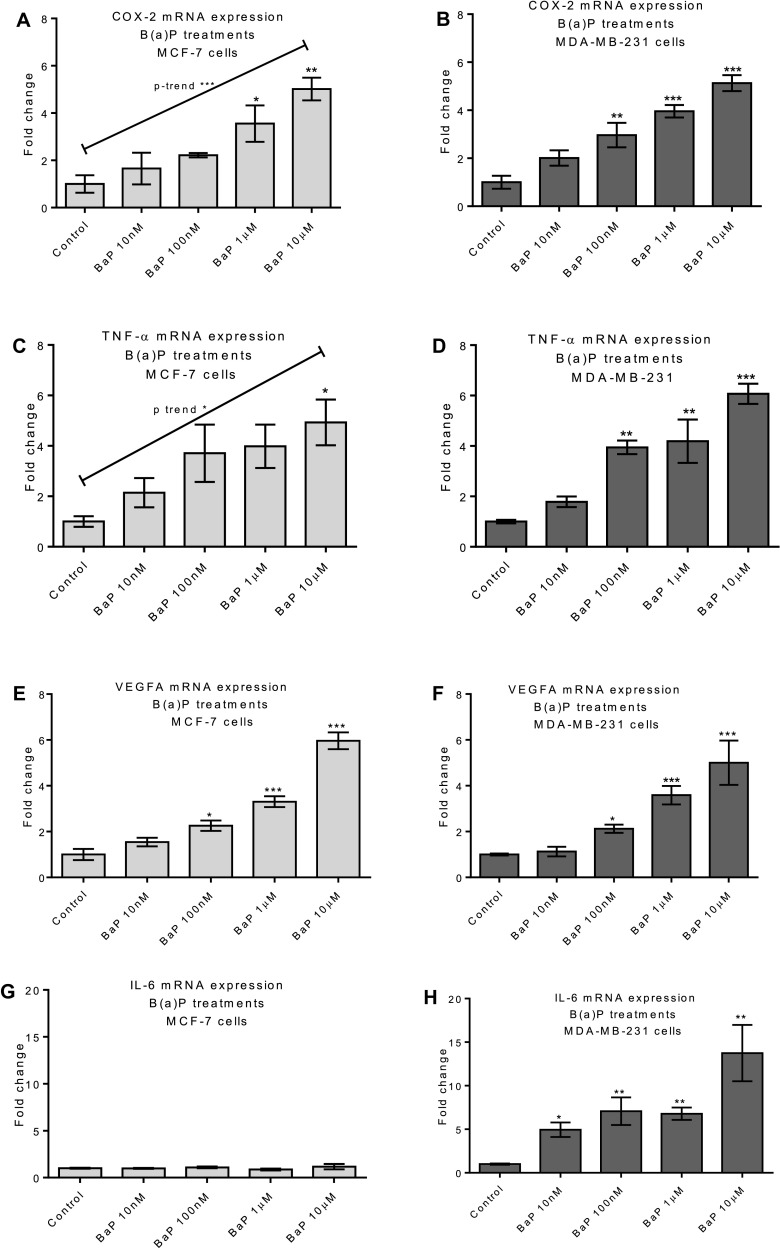
Regulation of inflammatory mediators by B(a)P in breast cells; MCF-7 (**a, c, e, g**) and MDA-MB-231 (**b, d, f, h**) cells. Inflammation modulators were measured by RT-qPCR; COX-2 (**a, b**); TNF-α (**c, d**); VEGFA (**e, f**); IL-6 (**g, h**). Cells were treated with B(a)P for 6 h. DMSO (0.1%) was used as a negative control. Data were normalized to mRNA expression of GAPDH and are shown relative to DMSO control. Statistically significant differences were calculated using one-way ANOVA with a Dunnett post-test (GraphPad Prism 5) (***p < 0.001, **p < 0.01, *p < 0.05). Data are presented as a mean of at least three independent cultures. Error bars represent the S.E.M.

Treatment of cells with B(a)P for 48 h did not induce excessive cytotoxicity (see Table 1) in either cell line. A statistically significant cytotoxic effect was only noted at the highest dose of B(a)P used (10 µM), this being a 6.5% and 6% reduction in cell viability in MCF-7 and MDA-MB-231 cells, respectively. This very low level of cytotoxicity is unlikely to contribute to downstream phenotypic responses. A dose-dependent increase in both COX-2 and TNF-α expression was seen following B(a)P treatment in both cell lines (Fig. 1a–d). The response to B(a)P was less pronounced in MCF-7 cells and statistical significance was only achieved at the highest concentration of B(a)P employed; however, there was a statistically significant trend detected for both COX-2 and TNF-α (Fig. 1a, c). Additionally, VEGF-A mRNA expression was increased in a dose-dependent manner in both cell lines (Fig. 1e, f). However, upregulation of IL-6 mRNA expression was only observed in MDA-MB-231 cells and not in MCF-7 cells (Fig. 1g, h).

**Table 1:** Cytotoxicity, proliferation and migration data following treatment with different concentrations of B(a)P

Treatment	Cytotoxicity^a^		Proliferation^b^		Migration^c^	
	MCF-7	MDA-MB-231	MCF-7	MDA-MB-231	MCF-7	MDA-MB-231
DMSO control	90 ± 2.7	88.3 ± 1.2	40.0 ± 13.3	123.5 ± 34.6	20.7 ± 3.5	33.2 ± 2.8
B(a)P 10 nM	90.1 ± 2.3	87.3 ± 1.7	106.6 ± 74.4	189.3 ± 82.9	30.6 ± 6.5	40.5 ± 9.5
B(a)P 100 nM	87.2 ± 3.9	87.3 ± 2.3	101.6 ± 26.2	283.7 ± 69.1*	40.7 ± 9.5**	51.0 ± 3.4*
B(a)P 1 µM	85.9 ± 2.3	86.1 ± 4.9	137.3 ± 29.5*	342.9 ± 43.9**	50.6 ± 3.6***	68.2 ± 6.6***
B(a)P 10 µM	83.5 ± 1.0*	82.4 ± 0.7*	185.8 ± 13.3**	306.3 ± 27.4**	68.0 ± 6.5***	83.0 ± 1.4***

Data are presented as the mean of three independent cultures (mean ± SEM, *n* = 3). Statistically significant differences between B(a)P vs control were calculated using one-way ANOVA with a Dunnett post-test (GraphPad Prism 5) (***p < 0.001, **p < 0.01, *p < 0.05)

^a^% viable cells after 48 h of culture

^b^% increase in proliferation after 96 h of culture

^c^% migration after 72 h of culture using a wound assay

**Fig. 2:**
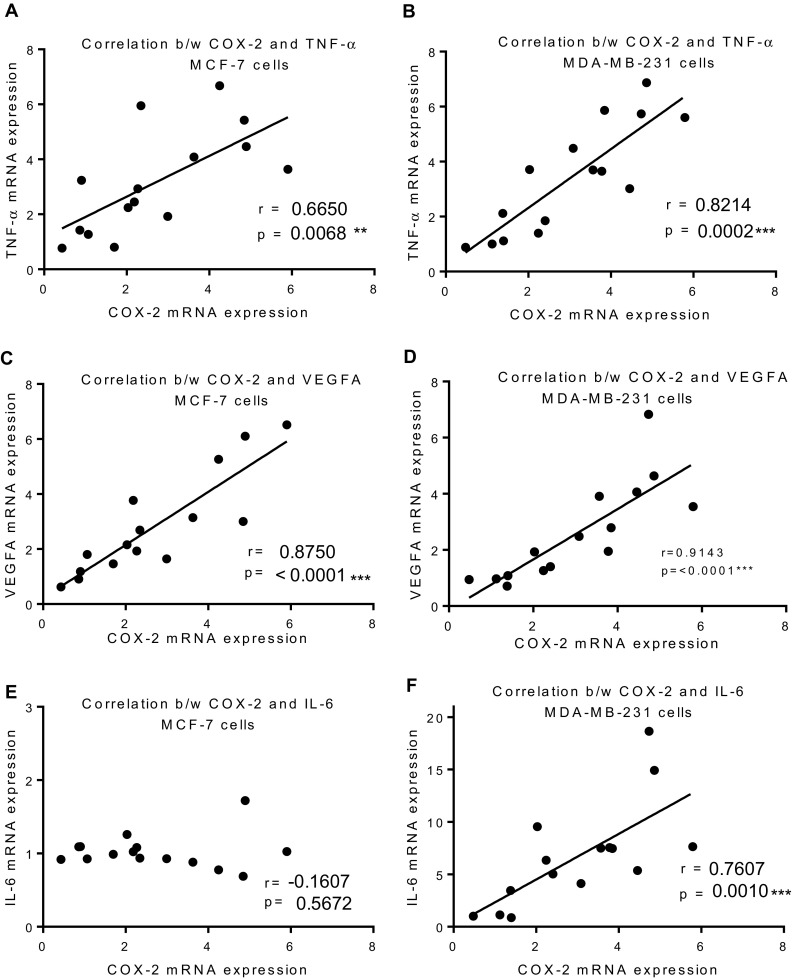
Correlation between different inflammation mediators in breast cells treated with B(a)P. Correlation between COX-2 and TNF-α in MCF-7 (**a**) and MDA-MB-231 (**b**), COX-2 and VEGFA in MCF-7 (**c**) and MDA-MB-231 (**d**), IL-6 and COX-2 in MCF-7 (**e**) and MDA-MB-231 (**f**). Pearson correlation coefficient test (*r*) was used for correlation analysis and statistical significance is shown as ***p < 0.001, **p < 0.01

TNF-α is a known inducer of COX-2 (Bansal et al. 2009; Lin et al. 2004; Mark et al. 2001) while VEGF is a downstream target of COX-2 (Toomey et al. 2009; Wu et al. 2006), suggesting that B(a)P can activate TNF-α leading to upregulation of COX-2 and subsequently VEGF. Consistent with this, a statistically significant level of correlation was found between TNF- α and COX-2 mRNA expression in B(a)P-treated MCF-7 and MDA-MB-231 cells (Fig. 2a, b). Additionally, a highly significant correlation was found between COX-2 and VEGF-A in both cell lines (Fig. 2c, d). Since production of pro-inflammation cytokines (IL-6, IL-1) has been shown to be responsible for the upregulation of COX-2 in tissue and various cell lines (Maihofner et al. 2003; Samad et al. 2001), the relationship between COX-2 and IL-6 was examined and found to be highly correlated in MDA-MB-231 cells but not in MCF-7 cells (Fig. 2e, f), suggesting IL-6 is involved in COX-2 upregulation in MDA-MB-231 cells but not MCF-7 cells (Fig. 2e, f).

**Fig. 3:**
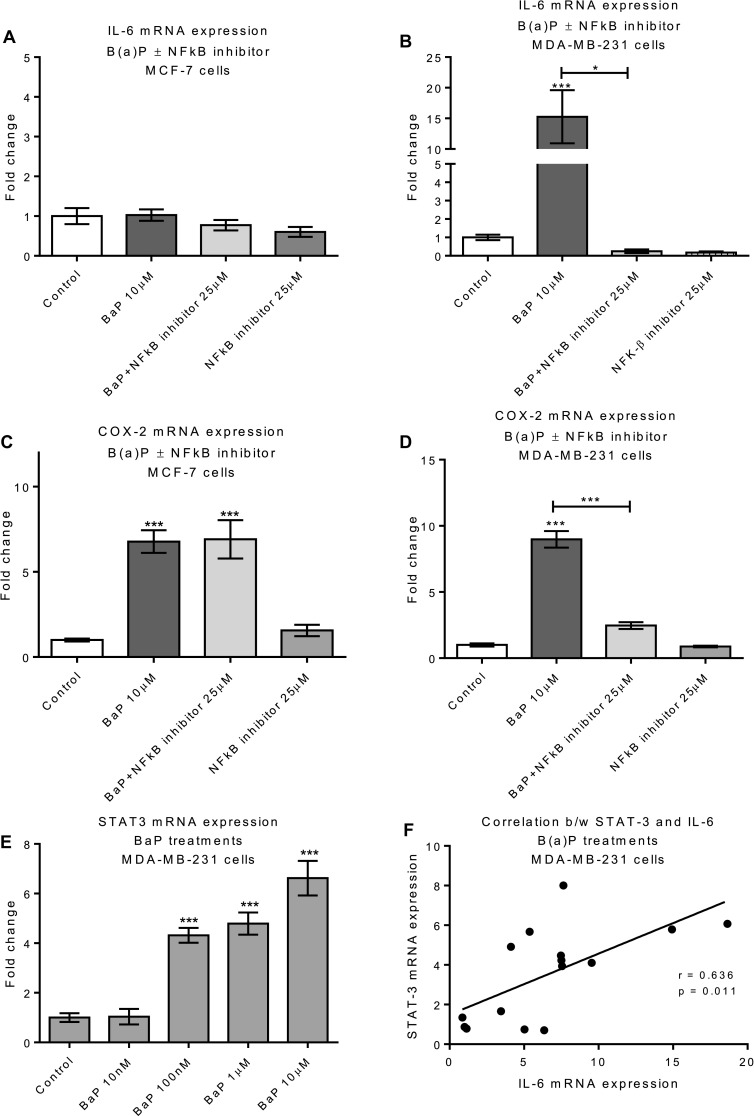
Effect of B(a)P and NFκB inhibition on IL-6, COX-2 and STAT3 expression. Cells were pretreated with 25 µM NFκB inhibitor for 24 h followed by 6 h B(a)P treatment and IL-6 and COX-2 mRNA expression was determined by qPCR in MCF-7 (**a, c**) and MDA-MB-231 (**b, d**). The effect of B(a)P on STAT3 expression in MDA-MB-231 cells (**e**) was assessed after 6 h of B(a)P treatment. Data were normalized to mRNA expression of GAPDH and are shown relative to control (0.1% DMSO). Statistical difference between two groups (B(a)P vs B(a)P + NFκB inhibitor) was determined by student *t* test. Statistically significant differences were calculated using one-way ANOVA with a Dunnett post-test (GraphPad Prism 5) (***p < 0.001, **p < 0.01, *p < 0.05). Data are presented as a mean of at least three independent cultures. Error bars represent the S.E.M. **f** Correlation between STAT3 and IL-6 mRNA expression in B(a)P-treated MDA-MB-231 cells. Pearson correlation coefficient test was used for correlation analysis (*p < 0.05)

IL-6 is a downstream target of NFκB (Brasier 2010; Domingo-Domenech et al. 2006; Liu et al. 2005), suggesting that NFκB pathway could be involved in IL-6 induction. Therefore, MCF-7 and MDA-MB-231 cells were treated with NFκB inhibitor (25 µM) for 24 h then with B(a)P for 6 h and assayed for IL-6 expression. In the presence of NFκB inhibitor, there was a significant down-regulation of B(a)P-induced IL-6 induction (Fig. 3b) in MDA-MB-231 cells. There was no significant effect of NFκB inhibitor on IL-6 mRNA expression in MCF-7 cells (Fig. 3a) nor was B(a)P exposure able to induce IL-6 expression, consistent with our data shown in Fig. 1g.

Previous studies have shown that NFκB mediates COX-2 expression (Charalambous et al. 2003, 2009; Chen et al. 2000; Lin et al. 2004; Maihofner et al. 2003); here we show that NFκB inhibitor (25 µM) substantially blocked the induction of COX-2 mRNA expression in MDA-MB-231 cells but not in MCF-7 cells (Fig. 3c, d), suggesting a selective role for NFκB in COX-2 induction in MDA-MB-231 cells. Overall, the results suggests that B(a)P exposure leads to activation of a TNF-α → COX-2 → VEGFA signaling pathway in both cell lines and selective involvement of NFκB and IL-6 only in MDA-MB-231 cells.

STAT-3 is a known downstream target of IL-6 (Patel et al. 2014); therefore, we considered the possibility that B(a)P could up-regulate STAT-3. MDA-MB-231 cells were, therefore, treated with B(a)P (6 h) and STAT-3 mRNA expression was examined by qPCR. Treatments with B(a)P significantly (*p* < 0.0001) up-regulated STAT3 mRNA expression in a dose-dependent manner (Fig. 3e) and a statistically significant correlation (*p* = 0.0109) was found between IL-6 and STAT-3 mRNA expression (Fig. 3f), supporting the observation that in MDA-MB-231 cells IL-6 can increase the expression of its downstream target STAT3.

### Can B(a)P increase the metastatic potential of breast cancer cells?

**Fig. 4:**
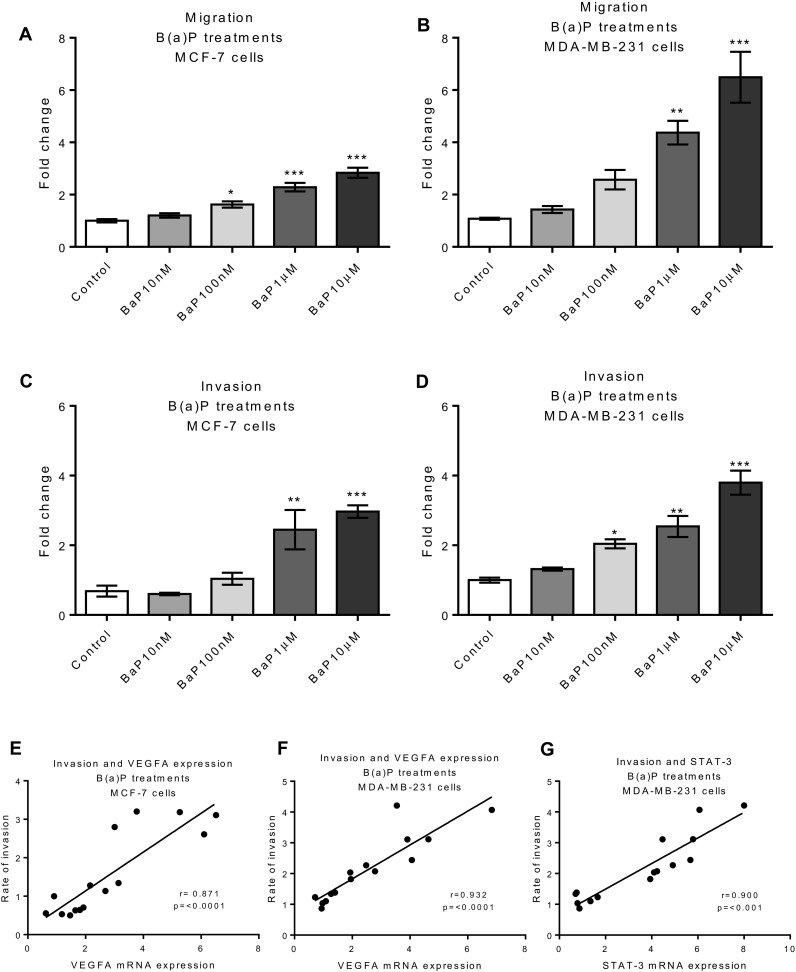
Effect of B(a)P on the migration and invasion of breast cells. Cells were treated with B(a)P and migration and invasion was observed after 72 h in MCF-7 (**a, c**) and MDA-MB-231 (**b, d**) cells. Migration and invasion was determined by transwell migration assay. Vehicle control was 0.1% DMSO. Statistically significant differences were calculated using one-way ANOVA with a Dunnett post-test (GraphPad Prism 5) (***p < 0.001, **p < 0.01, *p < 0.05). Data are presented as a mean of at least three biological replicates. Error bars represent the SEM for independent cultures (*n* = 3). Correlation between VEGFA and rate of invasion in MCF-7 (**e**) and MDA-MB-231 (**f**), STAT3 and rate of invasion in MDA-MB-231 (**g**). Pearson correlation coefficient test was used for correlation analysis. (0.0001–0.001 = ***p)

Metastatic potential is dependent on cell proliferation, migration and invasion. As COX-2, TNF-α, VEGF-A and IL-6 are pro-metastatic and B(a)P upregulates these mediators, it is possible that B(a)P can increase breast cancer cell metastasis. We determined that B(a)P dose-dependently increased proliferation of both MCF-7 and MDA-BA-231 cells, with a more potent proliferative effect on the latter (see Table 1). We then used a wound assay to assess the effect of B(a)P treatment on cell migration and found a consistent dose-dependent trend for increased cell migration in both cell lines (see Table 1). Migration induced by B(a)P-treated MCF-7 and MDA-MB-231 cells was also determined by transwell migration assay. Consistent with the results of the wound assay, a statistically significant level of induction (*p* < 0.001) in migration was observed following B(a)P treatment in both cell lines, with a stronger response in the MDA-MB-231 cells (Fig. 4a, b). Additionally, we examined the effect of B(a)P treatment using an invasion assay with the transwell insert coated with matrigel and cells must digest through the matrigel to migrate to the lower chamber. B(a)P induced invasion in both cell lines in a dose-dependent manner (Fig. 4c, d).

Since the expression of VEGFA and STAT-3 is thought to be involved in activation of the migratory and invasive potential of tumorigenic cells, we examined correlations between expression of these genes and invasive activity in response to B(a)P treatment. In the case of MCF-7 cells, there was a good correlation between VEGF expression and invasion, and with MDA-MB-231 cells expression of both VEGFA and STAT-3 strongly correlated with cell invasion (Fig. 4e–g, respectively).

### Can B(a)P regulate miRNAs via IL-6 upregulation in MDA-MB-231 cells?

**Fig. 5:**
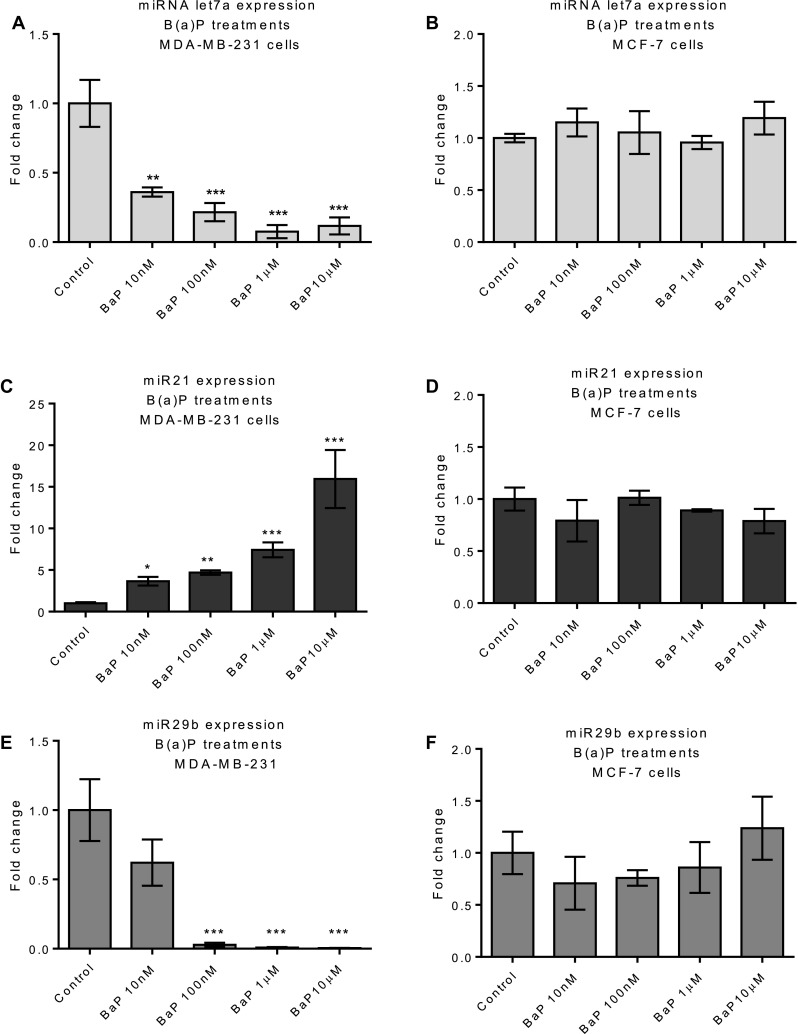
B(a)P effect on miRNA expression. Cells were treated with B(a)P for 6 h and miRNA expression was determined by qPCR in MDA-MB-231 (**a, c, e**) and MCF-7 (**b, d, f**). Vehicle control was 0.1% DMSO. Statistically significant differences were calculated using one-way ANOVA with a Dunnett post-test (GraphPad Prism 5) (***p < 0.001, **p < 0.01, *p < 0.05). Data are presented as a mean of at least three biological replicates. Error bars represent the SEM for independent cultures (*n* = 3)

Having shown that B(a)P can induce IL-6 expression, we hypothesized that the IL-6-mediated microRNAs let7a, miR21 and miR29b (Patel and Gooderham 2015) would be affected. B(a)P down-regulated let7a expression in a dose-dependent manner in MDA-MB-231 cells but not in MCF-7 (Fig. 5a, b). In contrast, a dose-related increase in tumor-promoting miR21 was observed after treatment with B(a)P in MDA-MB-231 cells but had little effect on MCF-7 cells (Fig. 5c, d). Like its effect on let7a expression, B(a)P treatment led to significant down-regulation of miR29b expression in MDA-MB-231 cells, but not in MCF-7 cells (Fig. 5e, f). Overall, B(a)P treatment altered expression of these three miRNAs in MDA-MB-231 but no significant changes were noted in the MCF-7 cells.

**Fig. 6:**
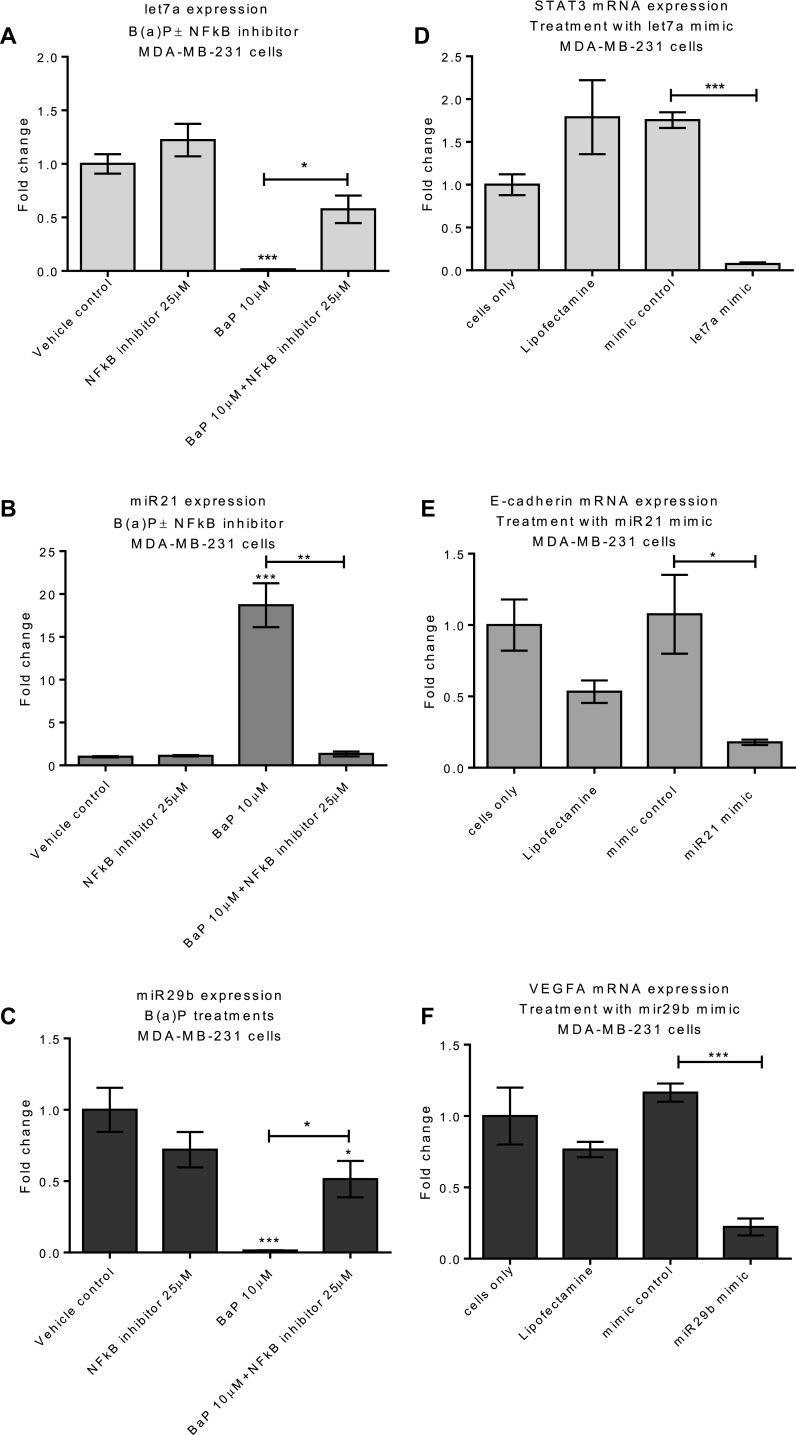
MiRNA expression and regulation of oncogenic targets: MDA-MB-231 cells were pretreated with NFκB inhibitor for 24 h followed by 6 h B(a)P treatment. MiRNA expression was determined by qPCR (**a**–**c**). Data were normalized to expression of U6 and is shown relative to vehicle control (0.1% DMSO). MDA-MB-231 cells were transfected with let7a, miR21, miR29b and the corresponding targets STAT3, e-cadherin and VEGFA were assessed, respectively (**d**–**f**). Statistically significant difference between mimic control and let7a/miR21/miR29b mimic target was determined by student *t* test. (GraphPad Prism 5) (***p < 0.001, **p < 0.01, *p < 0.05). Data are presented as a mean of at least three independent cultures. Error bars represent the SEM (*n* = 3)

Since let7a, miR21 and miR29b are all IL-6 regulated (Patel and Gooderham 2015), we determined whether the altered expression of miRNAs by B(a)P in MDA-MB-231 cells can be attributed to the ability of B(a)P to induce IL-6 expression in these cells. To confirm this, expression of these miRNAs was probed after blocking IL-6 upregulation using the NFκB inhibitor. MDA-MB-231 cells were treated for 24 h with NFκB inhibitor (25 µM) and subsequently with B(a)P (10 µM, 6 h) or NFκB inhibitor alone or B(a)P alone. As expected, B(a)P (10 µM) alone significantly reduced the let7a and miR29b expression; however, pre-treatment with NFκB inhibitor attenuated the B(a)P-mediated suppression (Fig. 6a, c). Treatment with NFκB alone did not alter basal expression of let7a and miR29b. In contrast, B(a)P alone significantly induced expression of miR21, but this induction was reduced to basal levels of expression by pre-treatment of cells with NFκB inhibitor (Fig. 6b). Collectively, these results support a role for NFκB in the regulation of the miRNA response in MDA-MB-231 cells treated with B(a)P.

### Can B(a)P-mediated let7a, miR29b and miR21 expression influence oncogenic targets in MDA-MB-231 cells?

As shown in Fig. 3e, B(a)P upregulates STAT3 in MDA-MB-231 cell line; STAT3 plays an important role in supporting tumor growth. We have also shown that B(a)P down-regulates Let7a (Fig. 5a) and it is reported that STAT3 is a target for let7a (Meng et al. 2007); therefore, regulation of STAT3 by let7a was investigated in our MDA-MB-231 cell model. Cells were transfected with let7a mimic and STAT3 mRNA expression was examined (Fig. 6d). Overexpression of let7a (30-fold compared to basal expression as determined by qPCR) significantly (*p* < 0.0001) decreased STAT3 mRNA expression (Fig. 6d), confirming the let7a regulation of pro-metastatic STAT3 expression in our MDA-MB-231 cell model. This is consistent with B(a)P regulating STAT3 expression via NFκB, IL-6 and let7a pathways.

The microRNA miR21 is reported to affect cell motility and stimulate epithelial–mesenchymal transition (EMT) (Cao et al. 2016). We, therefore, considered the possibility that upregulation of miR21 by B(a)P in our MDA-MB-231 cell model could down-regulate E-cadherin to potentiate the invasiveness of MDA-MB-231 cells. MDA-MB-231 cells transfected with miR21 mimic for 48 h (51-fold increase in miR21 expression compared to basal expression, confirmed by qPCR) significantly decreased E-cadherin mRNA expression (*p* = 0.0365) (Fig. 6e).

Our studies further show that B(a)P can down-regulate miR29b expression (Fig. 5e) and upregulate VEGFA (Fig. 1f). VEGF mRNA is a reputed target for miR29b (Zhang et al. 2014); therefore, we investigated the effect of miR29b overexpression on VEGF levels. Using a miR29b mimic, we achieved ~ 20-fold induction of miR29b expression compared to basal levels (confirmed by qPCR) in our MDA-MB-231 cell model and a corresponding reduction in VEGFA mRNA expression (Fig. 6f), consistent with the inverse relationship between miR29b and VEGFA.

### Effect of let7a, miR21 and miR29b expression on breast cancer cell metastatic behavior

We have shown that B(a)P can alter the expression of let7a, miR21 and miR29b and these three miRNAs in turn can regulate various tumor-promoting oncogenic targets. Therefore, we investigated the effect of these miRNAs on the metastatic behavior of breast cancer cells using miRNA mimics (overexpression) and transwell migration/invasion assays.

**Fig. 7:**
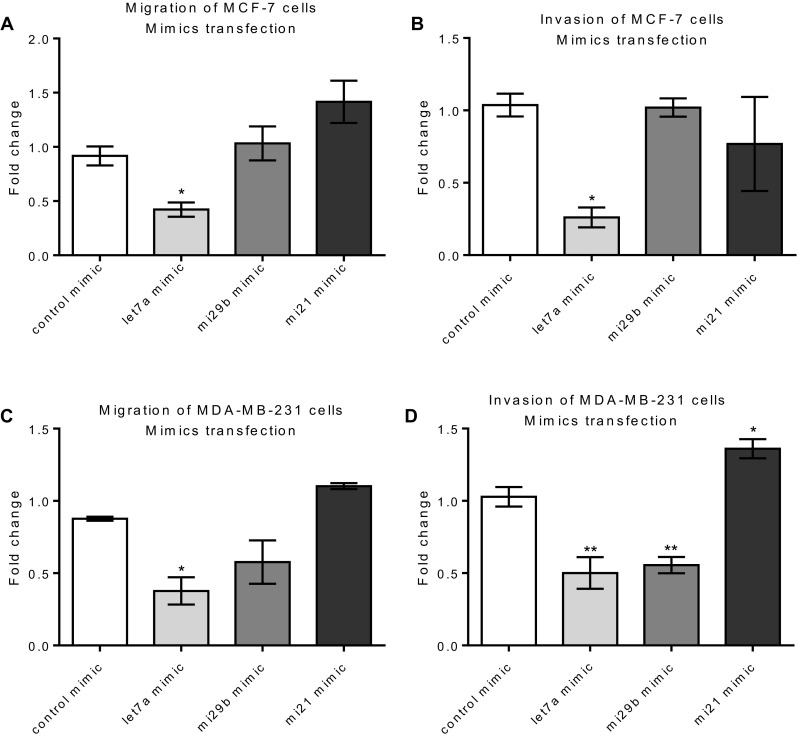
Effect of let7a, miR29b and miR21 expression on migration and invasion of MCF-7 and MDA-MB-231 cells. Let7a, miR21 and miR29b mimics were transfected into MCF-7 (**a, b**) and MDA-MB-231 (**c, d**) cells. Transwell migration (**a, c**) and invasion (**b, d**) assays were performed over 72 h. Migrated cells were quantified using AlamarBlue. Significant differences were calculated using one-way ANOVA followed by a Dunnet post-test (GraphPad Prism 5, ***p < 0.001, **p < 0.01, *p < 0.05). Error bars represent the SEM for independent cultures (*n* = 3)

Cell migration/invasion was quantified 72 h post-treatment using the AlamarBlue assay. MCF-7 cells were transfected with let7a/miR29b/miR21 mimics for 24 h and intracellular expression confirmed by qPCR quantification as 75-, 28- and 40-fold induction over basal levels, respectively. Let7a overexpression reduced both migration and invasion of MCF-7 cells (Fig. 7a, b). No significant changes in the migration or invasion of MCF-7 cells was observed following treatment with miR29b and miR21 mimics (Fig. 7a, b), despite a 28- and 40-fold overexpression compared to basal levels, respectively (measured by qPCR) in the MCF-7 cells.

Using the MDA-MB-231 cell model, transfection of the Let7a, miRNA21 and miRNA29 mimics increased expression 25-, 50- and 22-fold, respectively (determined by qPCR) over the basal levels after 48 h. As with the MCF-7 model, Let7a significantly decreased the migration (*p* < 0.05) and invasion (*p* < 0.01) of MDA-MB-231 cells (Fig. 7c, d). Unlike the MCF-7 model, MiR29b mimic significantly decreased (*p* < 0.01) invasion but the effect was less pronounced and not significantly different from the control for the migration assay (Fig. 7c, d). In contrast, overexpression of miR21 increased the migration and invasion of MDA-MB-231 cells (Fig. 7c, d), consistent with a pro-metastatic potential in this cell line.

## Discussion

Exposure to environmental carcinogens is an established contributor to the incidence of breast cancers (David and Gooderham 2016; DeBruin and Josephy 2002; McPherson et al. 2000). The powerful mammary environmental carcinogen B(a)P is thought to primarily act as a genotoxic carcinogen, leading to DNA damage (Brooks et al. 1999; David et al. 2016; David and Gooderham 2016). However, exposure to B(a)P, in addition to the metabolic activation of DNA-damaging species, is known to invoke a wide range of biological responses, including nuclear receptor activation (primarily the Ah receptor), which can lead to gene expression changes (Zhu and Gooderham 2002; Zhu et al. 2005). Many of these biological responses initiate, promote, and support carcinogenesis.

Inflammation is one of these key drivers of the carcinogenic process. In this study, we have examined a number of genes known to be involved in the inflammation response and breast cancer metastasis following treatment with B(a)P. We have used two human mammary cell lines, MCF-7 and MDA-MB-231 cells, which differ in their estrogen receptor (ER) status being ER-α positive and ER-α negative, respectively.

COX-2, TNF-α, VEGF-A and IL-6 are tumorigenic genes that are elevated in breast cancer. COX-2 is an inflammation cytokine expressed in 40% of invasive breast tumors and has been linked with the increased metastasis and poor prognosis of breast cancer (Miller et al. 2005; Ristimaki et al. 2002; Singh et al. 2007). Tumor necrosis factor-alpha (TNF-α) is an inflammation cytokine and has been shown to increase the growth of breast cancer cells (Danforth and Sgagias 1996). VEGFA, a signal protein involved in angiogenesis, is a metastatic marker and elevated levels were detected in the serum of breast cancer patients (Byrne et al. 2007). IL-6 is a pro-inflammation cytokine and elevated levels are linked with poor prognosis of breast cancer (Lin et al. 2015).

**Fig. 8:**
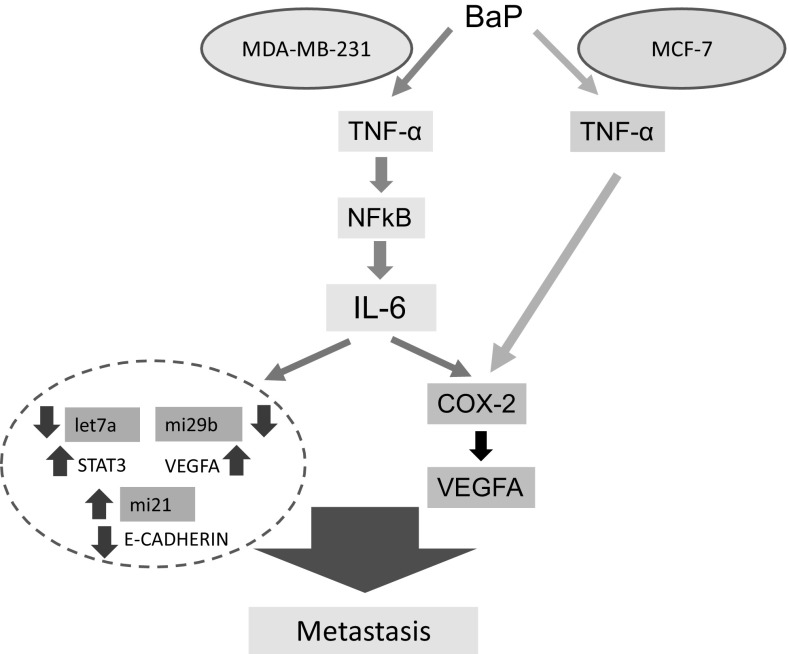
Regulation of inflammation mediators and miRNAs in breast cells. In MDA-MB-231 cells, B(a)P leads to the upregulation of IL-6 via TNF-α-NFκB, which further increases COX-2 and VEGFA expression to promote metastasis. B(a)P-mediated upregulation of IL-6 deregulates let7a, miR29b and miR21. In MCF-7 cells, B(a)P upregulates TNF-α and COX-2 to increase VEGFA expression to promote migration and invasion of MCF-7 cells

In this study, we show that B(a)P can activate an inflammation pathway involving TNF-α, COX-2 and VEGFA in two mammary cell lines; however, the details of the pathway are cell dependent. TNF-α has been shown to increase the activity of protein kinase C (PKC-alpha) and protein tyrosine kinase to activate NFκB, which is a COX-2 promoter (Chen et al. 2000). COX-2 expression is an indicator of aggressiveness and poor prognosis of breast cancer (Ashrafian et al. 2011; Jana et al. 2014; Nassar et al. 2007). We report that an NFκB inhibitor partially suppressed COX-2 mRNA expression in MDA-MB-231 cells but not in MCF-7 cells. This cell-specific divergence suggests that in MCF-7 cells TNF-α can regulate COX-2 mRNA expression by pathways independent of NFκB. This cell-specific pathway divergence is summarized in Fig. 8. We further show that B(a)P activation of NFκB also results in upregulation of IL-6. The mechanism whereby B(a)P selectively regulates IL-6 in MDA-MB-231 cells is, therefore, important to understand.

IL-6 upregulation by B(a)P was shown to be driven by NFκB yet only in MDA-MB-231 cells and not in MCF-7 cells. Significantly, we found that IL-6 is constitutively detectable in MDA-MB-231 cells but expression is very low in MCF-7 cells, consistent with a previous report (Faggioli et al. 1996), suggesting that expression of IL-6 in MDA-MB-231 cells is primed for induction. IL-6 increases the metastatic potential of cancer cells and its overexpression is linked with poor prognosis of breast cancer (Lin et al. 2015).

The difference in response between MCF-7 cells and MDA-MB-231 cells to B(a)P-mediated IL-6 upregulation is striking. The reason behind this differential cell response could be associated with ER-α status. It is also notable that MCF-7 cells are ER-α positive, whereas MDA-MB-231 cells are ER-α negative, and it has been shown that the estrogen receptor can interact with the NFκB receptor to inhibit the production of IL-6 in a hormone-dependent manner (Liu et al. 2005).

We are not aware of any published report of upregulation of IL-6 by B(a)P although previously B(a)P has been shown to increase the production of pro-inflammation IL-8 via NFκB activation in lung cancer A549 cell line (Pei et al. 2002). Like IL-6, IL-8 is up-regulated in breast cancer tissues compared to normal breast cells and is associated with poor prognosis (Lin et al. 2015; Singh et al. 2013).

COX-2 is a known downstream target of IL-6 (Anderson et al. 1996; Moon and Pestka 2003) and IL-6 can induce COX-2 leading to upregulation of matrix metalloproteinase-9 (MMP9), an endopeptidase that participates in various pathologic processes (Kothari et al. 2014). MMPs are known to be involved in cell invasion, through their proteolytic digestion of basement membrane thereby facilitating cell movement. This activity is key in metastasis, a multistage process that involves cell detachment, migration, invasion, proliferation and extravasation to distant sites (Stracke and Liotta 1992). Our mechanistic study shows that B(a)P can increase the metastatic potential of breast cancer cells by upregulating proliferation, migration and invasion in our cell-based model.

The ability of B(a)P to induce upregulation of inflammation pathways in mammary cancer cells and the associated proliferative, migration and invasion phenotype is consistent with B(a)P’s ability to initiate and promote mammary carcinogenesis. Phenotypic changes associated with the development of an inflammation environment, and particularly the involvement of IL-6, have been reported to involve dysregulation of microRNA (miRNA) (Patel and Gooderham 2015). Furthermore, dysregulation of miRNA is a common feature of many cancers including breast cancer (van Schooneveld et al. 2015).

The ability of B(a)P to induce IL-6 prompted our focus on microRNAs Let7a, miR21 and miR29b, all of which are IL-6-regulated (Patel and Gooderham 2015). We show that B(a)P can regulate let7a, miR29b and miR21 in MDA-MB-231 cells but less effectively in MCF-7 cells (Fig. 5) which may be attributable to the absence of IL-6 upregulation by B(a)P in these cells. This was confirmed by treatment with NFκB inhibitor that blocks B(a)P-mediated IL-6 increase. In line with this, an epigenetic feedback loop has been shown to exist between IL-6, NFκB and let7a (Iliopoulos et al. 2009).

Significantly, the dose-dependent decrease in miR29b expression detected in response to B(a)P was also associated with the decrease in the metastatic (invasion) potential of MDA-MB-231 cells, while no significant changes were seen in MCF-7 cells. Previous studies have shown that MiR29b negatively regulates migration by downregulating the expression of VEGF (Amodio et al. 2013; Zhang et al. 2014) and IL-8 mRNA expression (Amodio et al. 2013), and clinical studies have shown that miR29b exhibits tumor-suppressive effects in breast cancer (Qin et al. 2015; Shinden et al. 2015).

In the current study, B(a)P up-regulated miR21 expression and miR21 overexpression up-regulated the migration of MDA-MB-231 cells. Consistent with this observation, miR21 is an oncogenic miRNA that targets pro-apoptotic PTEN (phosphatase and tensin homolog) and PDCD4 (programmed cell death 4 gene) expression (Iliopoulos et al. 2010) and increases the migratory potential of a human keratinocyte cell line (Lu et al. 2015), nasopharyngeal carcinoma (Qiu et al. 2015), and colorectal cancer (Ferraro et al. 2014).

Activation of epithelial–mesenchymal transition (EMT) is a hallmark of metastatic cells (Thiery 2002; Voulgari and Pintzas 2009). E-Cadherin is a key adhesion molecule and its down-regulation is a classic marker of EMT (Thiery 2002). We have shown that B(a)P increased the migration and invasion of breast cells, up-regulated the expression of miR21, and down-regulated E-cadherin (in our MDA-MB-231 cell model). This is consistent with the recent reports that overexpression of miR21 can decrease E-cadherin expression and EMT (Ferraro et al. 2014; Liu et al. 2015) and the observation that cigarette smoke (a source of B(a)P) is shown to increase the EMT by the loss of E-cadherin in lung (Nagathihalli et al. 2012). Clinically, miR21 expression has been directly related to breast cancer metastasis and poor prognosis of breast cancer (Yan et al. 2008). Significantly, the B(a)P upregulation of miR21 expression and increased invasive propensity noted in ERα-negative mammary MDA-MB-231cells were not evident in the ERα-positive MCF-7 cells which potentially has clinical relevance.

Our finding that B(a)P can upregulate IL-6 and STAT3 expression and downregulate let7a expression is consistent with previous reports that Let7a expression is altered in response to IL-6 expression both in vivo and in vitro in cholangiocytes, and let7a can contribute to the phosphorylation of STAT3 via neurofibromatosis 2 (NF2) gene resulting in upregulation of STAT3 expression (Meng et al. 2007). This proposes that B(a)P increases IL-6 to decrease let7a expression, which leads to phosphorylation of STAT3. Furthermore, IL-6-mediated STAT3 upregulation leads to deregulation of miR21 with an inverse correlation between miR21 and E-cadherin. These pathways are summarized in Fig. 8.

Toxicological studies for safety assessment of compounds have traditionally focused on genotoxic responses leading to phenotypic change (Lizarraga et al. 2012); however, more recently, miRNAs have gained attention as negative regulators of mRNA expression (Lizarraga et al. 2012) bringing about phenotypic change via epigenetic mechanisms. Here, we show that traditional genotoxic agents such as B(a)P can also influence mRNA expression through non-genotoxic mechanisms involving microRNAs and these effects offer mechanistic insights into BaP-mediated chemical carcinogenesis.

Altered miRNA expression in response to B(a)P suggests that non-genotoxic events in addition to the well-established DNA-damaging abilities of B(a)P are important in B(a)P-driven phenotype changes leading to tumorigenicity. Furthermore, the regulation of miRNAs by B(a)P in MDA-MB-231 cells indicates cell selectivity in the response. Potentially, targeting these pathways can be of therapeutic value in a cell/tissue-specific manner. Overall, these data offer mechanistic support for the diverse biological properties of the environmental carcinogen B(a)P and reinforce the notion that the carcinogenicity of B(a)P is more than just its genotoxic potential. These data highlight the fact that environmental carcinogens can not only initiate the neoplastic process through mutation but can modulate the progression of cancer by regulating other biological events such as miRNA expression.

## References

[CR1] Amodio N, Bellizzi D, Leotta M (2013). miR-29b induces SOCS-1 expression by promoter demethylation and negatively regulates migration of multiple myeloma and endothelial cells. Cell cycle (Georgetown Tex).

[CR2] Anderson GD, Hauser SD, McGarity KL, Bremer ME, Isakson PC, Gregory SA (1996). Selective inhibition of cyclooxygenase (COX)-2 reverses inflammation and expression of COX-2 and interleukin 6 in rat adjuvant arthritis. J Clin Investig.

[CR3] Armstrong B, Doll R (1975). Environmental factors and cancer incidence and mortality in different countries, with special reference to dietary practices. Int J Cancer.

[CR4] Ashrafian H, Ahmed K, Rowland SP (2011). Metabolic surgery and cancer protective effects of bariatric procedures. Cancer.

[CR5] Baccarelli A, Bollati V (2009). Epigenetics and environmental chemicals. Curr Opin Pediatr.

[CR6] Balkwill F, Mantovani A (2001). Inflammation and cancer: back to Virchow?. Lancet.

[CR7] Bansal K, Narayana Y, Balaji KN (2009). Inhibition of TNF-alpha-induced cyclooxygenase-2 expression by Mycobacterium bovis BCG in human alveolar epithelial A549 cells. Scand J Immunol.

[CR8] Brasier AR (2010). The nuclear factor-kappaB-interleukin-6 signalling pathway mediating vascular inflammation. Cardiovasc Res.

[CR9] Brooks RA, Gooderham NJ, Edwards RJ, Boobis AR, Winton DJ (1999). The mutagenicity of benzo[a]pyrene in mouse small intestine. Carcinogenesis.

[CR10] Byrne GJ, McDowell G, Agarawal R, Sinha G, Kumar S, Bundred NJ (2007). Serum vascular endothelial growth factor in breast cancer. Anticancer Res.

[CR11] Cao J, Liu J, Xu R, Zhu X, Liu L, Zhao X (2016). MicroRNA-21 stimulates epithelial-to-mesenchymal transition and tumorigenesis in clear cell renal cells. Mol Med Rep.

[CR12] Charalambous MP, Maihofner C, Bhambra U, Lightfoot T, Gooderham NJ (2003). Upregulation of cyclooxygenase-2 is accompanied by increased expression of nuclear factor-kappa B and I kappa B kinase-alpha in human colorectal cancer epithelial cells. Br J Cancer.

[CR13] Charalambous MP, Lightfoot T, Speirs V, Horgan K, Gooderham NJ (2009). Expression of COX-2, NF-kappa B-p65, NF-kappa B-p50 and IKK alpha in malignant and adjacent normal human colorectal tissue. Br J Cancer.

[CR14] Chen CC, Sun YT, Chen JJ, Chiu KT (2000). TNF-alpha-induced cyclooxygenase-2 expression in human lung epithelial cells: involvement of the phospholipase C-gamma 2, protein kinase C-alpha, tyrosine kinase, NF-kappa B-inducing kinase, and I-kappa B kinase 1/2 pathway. J Immunol.

[CR15] Courter LA, Pereira C, Baird WM (2007). Diesel exhaust influences carcinogenic PAH-induced genotoxicity and gene expression in human breast epithelial cells in culture. Mutat Res.

[CR16] Coussens LM, Werb Z (2002). Inflammation and cancer. Nature.

[CR17] Cui Y, Miller AB, Rohan TE (2006). Cigarette smoking and breast cancer risk: update of a prospective cohort study. Breast Cancer Res Treat.

[CR18] Danforth DN, Sgagias MK (1996). Tumor necrosis factor alpha enhances secretion of transforming growth factor beta2 in MCF-7 breast cancer cells. Clin Cancer Res.

[CR19] David RM, Gooderham NJ (2016). Using 3D MCF-7 mammary spheroids to assess the genotoxicity of mixtures of the food-derived carcinogens benzo[a]pyrene and 2-amino-1-methyl-6-phenylimidazo[4,5-b]pyridine. Toxicol Res.

[CR20] David R, Ebbels T, Gooderham N (2016). Synergistic and antagonistic mutation responses of human MCL-5 cells to mixtures of benzo[a]pyrene and 2-amino-1-methyl-6-phenylimidazo[4,5-b]pyridine: dose-related variation in the joint effects of common dietary carcinogens. Environ Health Perspect.

[CR21] DeBruin LS, Josephy PD (2002). Perspectives on the chemical etiology of breast cancer. Environ Health Perspect.

[CR22] Domingo-Domenech J, Oliva C, Rovira A (2006). Interleukin 6, a nuclear factor-kappaB target, predicts resistance to docetaxel in hormone-independent prostate cancer and nuclear factor-kappaB inhibition by PS-1145 enhances docetaxel antitumor activity. Clin Cancer Res.

[CR23] Faggioli L, Costanzo C, Merola M (1996). Nuclear factor kappa B (NF-kappa B), nuclear factor interleukin-6 (NFIL-6 or C/EBP beta) and nuclear factor interleukin-6 beta (NFIL6-beta or C/EBP delta) are not sufficient to activate the endogenous interleukin-6 gene in the human breast carcinoma cell line MCF-7. Comparative analysis with MDA-MB-231 cells, an interleukin-6-expressing human breast carcinoma cell line. Eur J Biochem.

[CR24] Ferraro A, Kontos CK, Boni T (2014). Epigenetic regulation of miR-21 in colorectal cancer: ITGB4 as a novel miR-21 target and a three-gene network (miR-21-ITGΒ4-PDCD4) as predictor of metastatic tumor potential. Epigenetics.

[CR25] Gooderham NJ, Koufaris C (2014). Using microRNA profiles to predict and evaluate hepatic carcinogenic potential. Toxicol Lett.

[CR26] Guo J, Xu Y, Ji W, Song L, Dai C, Zhan L (2015). Effects of exposure to benzo[a]pyrene on metastasis of breast cancer are mediated through ROS-ERK-MMP9 axis signaling. Toxicol Lett.

[CR27] Iliopoulos D, Hirsch HA, Struhl K (2009). An epigenetic switch involving NF-κB, Lin28, let-7 microRNA, and IL6 links inflammation to cell transformation. Cell.

[CR28] Iliopoulos D, Jaeger SA, Hirsch HA, Bulyk ML, Struhl K (2010). STAT3 activation of miR-21 and miR-181b-1 via PTEN and CYLD are part of the epigenetic switch linking inflammation to cancer. Mol cell.

[CR29] Jana D, Sarkar DK, Ganguly S (2014). Role of cyclooxygenase 2 (COX-2) in prognosis of breast cancer. Indian J Surg Oncol.

[CR30] Kothari P, Pestana R, Mesraoua R (2014). IL-6-mediated induction of matrix metalloproteinase-9 is modulated by JAK-dependent IL-10 expression in macrophages. J Immunol.

[CR31] Koufaris C, Wright J, Currie RA, Gooderham NJ (2012). Hepatic MicroRNA profiles offer predictive and mechanistic insights after exposure to genotoxic and epigenetic hepatocarcinogens. Toxicol Sci.

[CR32] Lee J, Taneja V, Vassallo R (2012). Cigarette smoking and inflammation: cellular and molecular mechanisms. J Dent Res.

[CR33] Ley SH, Sun Q, Willett WC (2014). Associations between red meat intake and biomarkers of inflammation and glucose metabolism in women. Am J Clin Nutr.

[CR34] Li H, Fan X, Houghton J (2007). Tumor microenvironment: the role of the tumor stroma in cancer. J Cell Biochem.

[CR35] Lin CC, Hsiao LD, Chien CS, Lee CW, Hsieh JT, Yang CM (2004). Tumor necrosis factor-alpha-induced cyclooxygenase-2 expression in human tracheal smooth muscle cells: involvement of p42/p44 and p38 mitogen-activated protein kinases and nuclear factor-kappaB. Cell Signal.

[CR36] Lin CC, Lee IT, Yang YL, Lee CW, Kou YR, Yang CM (2010). Induction of COX-2/PGE(2)/IL-6 is crucial for cigarette smoke extract-induced airway inflammation: role of TLR4-dependent NADPH oxidase activation. Free Radic Biol Med.

[CR37] Lin S, Gan Z, Han K, Yao Y, Min D (2015). Interleukin-6 as a prognostic marker for breast cancer: a meta-analysis. Tumori.

[CR38] Liu H, Liu K, Bodenner DL (2005). Estrogen receptor inhibits interleukin-6 gene expression by disruption of nuclear factor κB transactivation. Cytokine.

[CR39] Liu Z, Jin Z-Y, Liu C-H, Xie F, Lin X-S, Huang Q (2015). MicroRNA-21 regulates biological behavior by inducing EMT in human cholangiocarcinoma. Int J Clin Exp Pathol.

[CR40] Lizarraga D, Gaj S, Brauers KJ, Timmermans L, Kleinjans JC, van Delft JH (2012). Benzo[a]pyrene-induced changes in microRNA-mRNA networks. Chem Res Toxicol.

[CR41] Lu X, Luo F, Liu Y (2015). The IL-6/STAT3 pathway via miR-21 is involved in the neoplastic and metastatic properties of arsenite-transformed human keratinocytes. Toxicol Lett.

[CR42] Maihofner C, Charalambous MP, Bhambra U, Lightfoot T, Geisslinger G, Gooderham NJ (2003). Expression of cyclooxygenase-2 parallels expression of interleukin-1beta, interleukin-6 and NF-kappaB in human colorectal cancer. Carcinogenesis.

[CR43] Mark KS, Trickler WJ, Miller DW (2001). Tumor necrosis factor-alpha induces cyclooxygenase-2 expression and prostaglandin release in brain microvessel endothelial cells. J Pharmacol Exp Therap.

[CR44] Mbeunkui F, Johann DJ (2009). Cancer and the tumor microenvironment: a review of an essential relationship. Cancer Chemother Pharmacol.

[CR45] McPherson K, Steel CM, Dixon JM (2000). ABC of breast diseases. Breast cancer-epidemiology, risk factors, and genetics. BMJ.

[CR46] Meng F, Henson R, Wehbe-Janek H, Smith H, Ueno Y, Patel T (2007). The MicroRNA let-7a modulates interleukin-6-dependent STAT-3 survival signaling in malignant human cholangiocytes. J Biol Chem.

[CR47] Miller ME, Holloway AC, Foster WG (2005). Benzo-[a]-pyrene increases invasion in MDA-MB-231 breast cancer cells via increased COX-II expression and prostaglandin E2 (PGE2) output. Clin Exp Metastasis.

[CR48] Moon Y, Pestka JJ (2003). Cyclooxygenase-2 mediates interleukin-6 upregulation by vomitoxin (deoxynivalenol) in vitro and in vivo. Toxicol Appl Pharmacol.

[CR49] Nagathihalli NS, Massion PP, Gonzalez AL, Lu P, Datta PK (2012). Smoking induces epithelial-to-mesenchymal transition in non–small cell lung cancer through HDAC-mediated downregulation of E-cadherin. Mol Cancer Ther.

[CR50] Nassar A, Radhakrishnan A, Cabrero IA, Cotsonis G, Cohen C (2007). COX-2 expression in invasive breast cancer: correlation with prognostic parameters and outcome. Appl Immunohistochem Mol Morphol.

[CR51] Papaioannou MD, Koufaris C, Gooderham NJ (2014). The cooked meat-derived mammary carcinogen 2-amino-1-methyl-6-phenylimidazo[4,5-b]pyridine (PhIP) elicits estrogenic-like microRNA responses in breast cancer cells. Toxicol Lett.

[CR52] Patel SA, Gooderham NJ (2015). IL6 Mediates Immune and Colorectal Cancer Cell Cross-talk via miR-21 and miR-29b. Mol Cancer Res.

[CR53] Patel SA, Bhambra U, Charalambous MP (2014). Interleukin-6 mediated upregulation of CYP1B1 and CYP2E1 in colorectal cancer involves DNA methylation, miR27b and STAT3. Br J Cancer.

[CR54] Pei XH, Nakanishi Y, Inoue H, Takayama K, Bai F, Hara N (2002). Polycyclic aromatic hydrocarbons induce IL-8 expression through nuclear factor kappaB activation in A549 cell line. Cytokine.

[CR55] Pufulete M, Battershill J, Boobis A, Fielder R (2004). Approaches to carcinogenic risk assessment for polycyclic aromatic hydrocarbons: a UK perspective. Regul Toxicol Pharmacol.

[CR56] Qin L, Li R, Zhang J, Li A, Luo R (2015). Special suppressive role of miR-29b in HER2-positive breast cancer cells by targeting Stat3. Am J Transl Res.

[CR57] Qiu F, Sun R, Deng N (2015). miR-29a/b enhances cell migration and invasion in nasopharyngeal carcinoma progression by regulating SPARC and COL3A1 gene expression. PLoS One.

[CR58] Ristimaki A, Sivula A, Lundin J (2002). Prognostic significance of elevated cyclooxygenase-2 expression in breast cancer. Cancer Res.

[CR59] Samad TA, Moore KA, Sapirstein A (2001). Interleukin-1[beta]-mediated induction of Cox-2 in the CNS contributes to inflammatory pain hypersensitivity. Nature.

[CR60] Shinden Y, Iguchi T, Akiyoshi S (2015). miR-29b is an indicator of prognosis in breast cancer patients. Mol Clin Oncol.

[CR61] Shivappa N, Sandin S, Lof M, Hebert JR, Adami HO, Weiderpass E (2015). Prospective study of dietary inflammatory index and risk of breast cancer in Swedish women. Br J Cancer.

[CR62] Singh B, Berry JA, Shoher A, Ayers GD, Wei C, Lucci A (2007). COX-2 involvement in breast cancer metastasis to bone. Oncogene.

[CR63] Singh JK, Simoes BM, Howell SJ, Farnie G, Clarke RB (2013). Recent advances reveal IL-8 signaling as a potential key to targeting breast cancer stem cells. Breast Cancer Res.

[CR64] Sonkoly E, Pivarcsi A (2011). MicroRNAs in inflammation and response to injuries induced by environmental pollution. Mutat Res.

[CR65] Stracke ML, Liotta LA (1992). Multi-step cascade of tumor cell metastasis. In Vivo.

[CR66] Thiery JP (2002). Epithelial-mesenchymal transitions in tumour progression. Nat Rev Cancer.

[CR67] Toomey DP, Murphy JF, Conlon KC (2009). COX-2, VEGF and tumour angiogenesis. Surgeon.

[CR68] van Schooneveld E, Wildiers H, Vergote I, Vermeulen PB, Dirix LY, Van Laere SJ (2015). Dysregulation of microRNAs in breast cancer and their potential role as prognostic and predictive biomarkers in patient management. Breast Cancer Res.

[CR69] van der Vaart H, Postma DS, Timens W, Ten Hacken NHT (2004). Acute effects of cigarette smoke on inflammation and oxidative stress: a review. Thorax.

[CR70] Voulgari A, Pintzas A (2009). Epithelial-mesenchymal transition in cancer metastasis: mechanisms, markers and strategies to overcome drug resistance in the clinic. Biochim et Biophys Acta.

[CR71] Wu G, Luo J, Rana JS, Laham R, Sellke FW, Li J (2006). Involvement of COX-2 in VEGF-induced angiogenesis via P38 and JNK pathways in vascular endothelial cells. Cardiovasc Res.

[CR72] Yan L-X, Huang X-F, Shao Q (2008). MicroRNA miR-21 overexpression in human breast cancer is associated with advanced clinical stage, lymph node metastasis and patient poor prognosis. RNA.

[CR73] Zhang K, Zhang C, Liu L, Zhou J (2014). A key role of microRNA-29b in suppression of osteosarcoma cell proliferation and migration via modulation of VEGF. Int J Clin Exp Pathol.

[CR74] Zhu H, Gooderham N (2002). Neoplastic transformation of human lung fibroblast MRC-5 SV2 cells induced by benzo[a]pyrene and confluence culture. Cancer Res.

[CR75] Zhu H, Smith C, Ansah C, Gooderham NJ (2005). Responses of genes involved in cell cycle control to diverse DNA damaging chemicals in human lung adenocarcinoma A549 cells. Cancer Cell Int.

